# Preparation of a Nanoscaled Poly(vinyl alcohol)/Hydroxyapatite/DNA Complex Using High Hydrostatic Pressure Technology for *In Vitro* and *In Vivo* Gene Delivery

**DOI:** 10.1155/2011/962743

**Published:** 2011-05-12

**Authors:** Tsuyoshi Kimura, Yoichi Nibe, Seiichi Funamoto, Masahiro Okada, Tsutomu Furuzono, Tsutomu Ono, Hidekazu Yoshizawa, Toshiya Fujisato, Kwangwoo Nam, Akio Kishida

**Affiliations:** ^1^Institute of Biomaterials and Bioengineering, Tokyo Medical and Dental University, 2-3-10 Kanda-Surugadai, Chiyoda-ku, Tokyo 101-0062, Japan; ^2^Japan Science and Technology Agency, CREST 5, Sanbancho, Chiyoda-ku, Tokyo 102-0075, Japan; ^3^Department of Biomedical Engineering, National Cardiovascular Center Research Institute, 5-7-1 Fujishirodai, Suita, Osaka 656-8565, Japan; ^4^Department of Environmental Chemistry and Materials, Okayama University, 3-1-1 Tsushimanaka, Okayama 700-8530, Japan; ^5^Department of Biomedical Engineering, Osaka Institute of Technology, 5-16-1 Omiya, Asahi-ku, Osaka 573-0196, Japan

## Abstract

Our previous research showed that poly(vinyl alcohol) (PVA) nanoparticles incorporating DNA with hydrogen bonds obtained by high hydrostatic pressurization are able to deliver DNA without any significant cytotoxicity. To enhance transfection efficiency of PVA/DNA nanoparticles, we describe a novel method to prepare PVA/DNA nanoparticles encapsulating nanoscaled hydroxyapatites (HAps) prepared by high hydrostatic pressurization (980 MPa), which is designed to facilitate endosomal escape induced by dissolving HAps in an endosome. Scanning electron microscopic observation and dynamic light scattering measurement revealed that HAps were significantly encapsulated in PVA/HAp/DNA nanoparticles. The cytotoxicity, cellular uptake, and transgene expression of PVA/HAp/DNA nanoparticles were investigated using COS-7 cells. It was found that, in contrast to PVA/DNA nanoparticles, their internalization and transgene expression increased without cytotoxicity occurring. Furthermore, a similar level of transgene expression between plasmid DNA and PVA/HAp/DNA nanoparticles was achieved using *in vivo* hydrodynamic injection. Our results show a novel method of preparing PVA/DNA nanoparticles encapsulating HAp nano-crystals by using high hydrostatic pressure technology and the potential use of HAps as an enhancer of the transfection efficiency of PVA/DNA nanoparticles without significant cytotoxicity.

## 1. Introduction

Polymeric gene delivery systems are of great interest in gene therapy because of their greater degree of safety compared to that of viral vectors. Many types of cationic polymers, such as poly-L-lysine and its derivatives [1, 2], polyethyleneimine [3], polyamidoamine dendrimer [4], and vinyl polymers [5], have been developed as gene carriers to aim at effective and safe *in vitro *and *in vivo* gene transfection into cells. They can spontaneously condense DNA by electrostatic interaction between positive charged groups of polycation and phosphate groups of DNA and form complexes, which are called polyplexes. The polyplex formation protects DNA from degradation by DNases in extracellular and intracellular pathways, resulting in the enhancement of gene transfection efficacy. However, the cytotoxicity of cationic polymers is an essential problem in the polyplex-based gene transfer field [6]. In addition, polymeric gene carriers may elicit nonspecific immune responses [7]. Therefore, significant efforts have been made towards decreasing the toxicity of polymeric gene carriers. 

Two main strategies have been proposed to address this issue. One is to attach polyethylene glycol (PEG), which is widely used as a nonionic, highly soluble, low toxicity polymer, to polymeric gene carriers, a process that is called “PEGylation.” PEGylation increases the water solubility of polyplexes and reduces the interaction of polyplex and serum and blood components, resulting in effective transfection without toxicity [8, 9]. The other is the use of non- or less cationic polymers, which can form complexes via nonelectrostatic interactions, such as hydrogen bonding. Double strand schizophyllan, which is one type of polysaccharide (*β*-1, 3 glucan), forms a triple helical complex with single-strand DNA through hydrogen bonding interaction [10]. Protective interactive noncondensing (PINC) polymers, poly (N-vinyl pyrrolidone) (PVP), and poly (vinyl alcohol) (PVA), form flexible polyplexes with DNA via hydrogen bonds [11, 12]. In addition, we have developed a novel formulation method of DNA complexes with nonionic, water-soluble polymers through hydrogen bonding interaction using high hydrostatic pressure technology. Under high hydrostatic pressure conditions, inter- and intramolecular hydrogen bonding interaction is effectively formed [13–15]. We previously reported that nanoscaled PVA/DNA complexes via hydrogen bonding interaction were obtained by high hydrostatic pressurization at 980 MPa and 40°C for 10 min [16]. The PVA/DNA nanoparticles were taken up by RAW264 cells with nontoxicity, and no significant gene expressions were observed. 

Traditionally, the calcium phosphate (Cap)-DNA coprecipitation method has been used for *in vitro* gene transfection because of CaP's biocompatibility, biodegradability, and ease of handling [17, 18]. Many CaP-DNA coprecipitation methods that particulate formation, being affected by pH [19], temperature [20], and buffer conditions [21], have been developed to aim at effective gene transfection. In addition, several researchers have proposed the idea of applying CaP-DNA coprecipitates produced in polyplexes to gene delivery. It is considered that polyplexes including CaP were internalized into cells through endocytosis pathways, in which the pH was lower than 5.5, and then the rupture of endosome and endosomal releases of polyplex were induced by osmotic shock [22, 23]. Currently, nanoscaled HAps, which are one of the forms of CaP, have been synthesized with well-controlled size and shape and utilized as gene carriers because of the capability of HAps to absorb DNA molecules [24]. 

On the basis of this background, in the current study, we used nanoscaled HAps (about 50 nm) as an endosomal escape reagent because of their ability to dissolve in endosome vesicles under low pH conditions. We investigated a method of preparing the PVA/DNA complexes encapsulating HAps by using high hydrostatic pressure technology in detail. Using the obtained PVA/HAp/DNA nanoparticles, the cellular uptake, cytotoxicity, and *in vitro* and *in vivo* transfection efficiency were examined to aim at effective and safe gene transfection.

## 2. Materials and Methods

### 2.1. Materials

PVA with a degree of polymerization of 1700 and a degree of saponification of 99.3% was kindly supplied from Kuraray Co. Ltd. (Osaka, Japan). HAp with an average diameter of 50 nm was synthesized by an emulsion system [25, 26] and then suspended in water. Plasmid DNA encoding a luciferase gene under an SV40 promoter (pGL3: 5.2 kbp) was purchased from Promega Co., Ltd., (Madison, USA).

### 2.2. Preparation of PVA/HA/pDNA Complexes

An aqueous PVA solution of 5 w/v% was prepared by autoclaving it three times for 30 min at 121°C and diluting it to various concentrations. An aqueous HAp suspension prepared by ultrasonication was added to the PVA solution. The DNA solution was mixed with the PVA/HAp suspension (final concentrations: PVA 0.001–1.0 w/v%, HA 0.0001–0.1 w/v%, DNA 0.0025 w/v%). The mixture solution of PVA, Hap, and DNA was hydrostatically pressurized at 980 MPa and 40°C for 10 min using a high hydrostatic pressure machine (Dr. Chef: Kobe steel, Kobe, Japan).

### 2.3. Characterization of PVA/HAp/DNA Complexes

The shapes of PVA/DNA (PVA: 1.0 w/v%) and PVA/HAp/DNA (PVA: 1.0 w/v%, HAp: 0.1 w/v%) complexes obtained by the high hydrostatic pressurization were observed with a scanning electron microscope (SEM, JSM-6301F, JEOL Co., Tokyo, Japan). One *μ*L of the complex solutions was dropped on a glass slide and dried in air. The sizes of the PVA/DNA and PVA/HAp/DNA complexes obtained by the high hydrostatic pressurization were measured by dynamic light scattering (DLS) using a Zetasizer Nano product (Malvern, Worcestershire, UK). The stability of DNA in PVA/DNA complex on 10% serum condition was investigated. The PVA/DNA complexes were incubated with medium containing 10% serum for 20 h. Then, they were subjected to in vitro transcription and translation system (TNT Quick coupled Transcription/Translation System, Promega Co., Ltd., Madison, USA), and the luciferase activity was measured by using an AB-2200 luminometer (ATTO, Corp., Tokyo, Japan) for 10 s.

### 2.4. Cytotoxicity of PVA/HAp/DNA Complexes

A mixture solution of PVA (2 w/v%) and HAp (0.2 w/v%) was prepared and diluted stepwise to 0.01 w/v% of PVA and 0.001 w/v% of HAp. An aqueous DNA solution of 0.005 w/v% was mixed with PVA/HAp mixtures for each concentration at an equal volume. Their mixtures were treated under 980 MPa at 40^*º*^C for 10 min using a high hydrostatic pressure machine. The COS-7 cells used were purchased from RIKEN Bioresource Center (BRC, Saitama, Japan). They were cultured in a complete modified eagle medium (DMEM, Life technologies Japan Ltd, Tokyo, Japan), supplemented with non-inactivated 10% fetal bovine serum (FBS), 50 IU/mL of penicillin, and 50 *μ*g/mL of streptomycin (ICN Biomaterials, Ohio, USA). The COS-7 cells (2.0 × 10^4^) on a 96-well plate were incubated with PVA/DNA and PVA/HAp/DNA complexes of various concentrations at 37°C for 20 h in the presence of FBS (10%). The cellular viability was assessed using a Cell Counting Kit-8 (Dojindo Laboratory, Tokyo, Japan) according to the manufacturer's instructions.

### 2.5. Cellular Uptake of PVA/HAp/DNA Complexes

The pGL3 plasmid DNA was labeled with rhodamine using a Label It kit (Panvera, Wis, USA) according to the manufacturer's instructions (Rh-DNA). HAp/Rh-DNA (HAp: 0.4 w/v%). PVA/Rh-DNA, and PVA/HAp/Rh-DNA complexes (PVA: 0.001 w/v%, HAp: 0.0001 w/v%) were added to COS-7 cells (1.0 × 10^5^) cultured in 24-well plates in the presence of FBS (10%), and incubated at 37°C for one and 24 h. After washing with PBS twice, the cells were observed under a fluorescent microscope.

### 2.6. In Vitro Transfection

COS-7 cells (8.0 × 10^4^) were cultured overnight in a 48-well plate. HAp/DNA (HAp: 0.4 w/v%), PVA/DNA, and PVA/HAp/DNA complexes (PVA: 0.001 w/v%, HAp: 0.0001 w/v%) were added to cells and incubated at 37°C for 24 h. The medium was removed from each well and washed with PBS twice. 50 *μ*L of a 1x luciferase cell culture lysis buffer (Promega Co., Ltd., Madison, USA) was added onto cells. For luciferase assay, 10 *μ*L of cell extract was mixed with 100 *μ*L of a luciferase assay reagent (Promega Co., Ltd., Madison, USA) and the luciferase activity was measured by using an AB-2200 luminometer (ATTO, Corp., Tokyo, Japan) for 10 s. The protein concentration of the supernatant was determined by using a DC protein assay kit (Bio-Rad laboratories, Inc., USA) according to the manufacturer's instructions.

### 2.7. In Vivo Transfection Using Hydrodynamic Injection Method

1.6 mL of the saline solution of PVA/DNA and PVA/HAp/DNA complexes (PVA: 0.001 w/v% or 0.01 w/v%, HAp: 0.0001 w/v% or 0.001 w/v%, DNA: 0.0025 w/v%) were prepared by high hydrostatic pressurization and injected by a hydrodynamic technique as previously described [27]. Briefly, mice were restrained, and the tail vein was accessed with a 25 gauge needle. Administration of the solution was performed in 10 seconds or less without extravasation; each group was represented by three or more animals. After 12, 24, and 72 h injection, the liver and lung were dissected from dead animals using the standard surgical procedures. 1 mL of lysis buffer (0.1 M Tris-HCl, 2 mM EDTA, and 0.1% Triton X-100, pH 7.8) was added to a piece of liver with wet weight of approximately 200 mg. The liver was homogenized for 15–20 s with a homogenizer (PT2100, KINEMATICA AG, Lucerne, Switzerland) at maximal speed, and the tissue homogenate was then centrifuged in a microcentrifuge for 10 min at 13000 g at 4°C. The protein concentration of the supernatant was determined by using a DC protein assay kit. For luciferase assay of the liver extract, the supernatant was further diluted 60-fold using an HEPES buffer. 10 *μ*L of supernatant of diluted liver extract was mixed with 100 *μ*L of luciferase assay reagent, and the luciferase activity was measured by using the AB-2200 luminometer for 10 s.

### 2.8. Statistical Analysis

All experiments were repeated at least three times (five times for DLS analysis), and the values are expressed as means ± standard deviations. Statistical analysis was performed using student's *t*-test, with the significant level set at *P* < .05.

## 3. Results and Discussion

### 3.1. Preparation and Characterization of PVA/HAp/DNA Complexes

**Figure 1:**
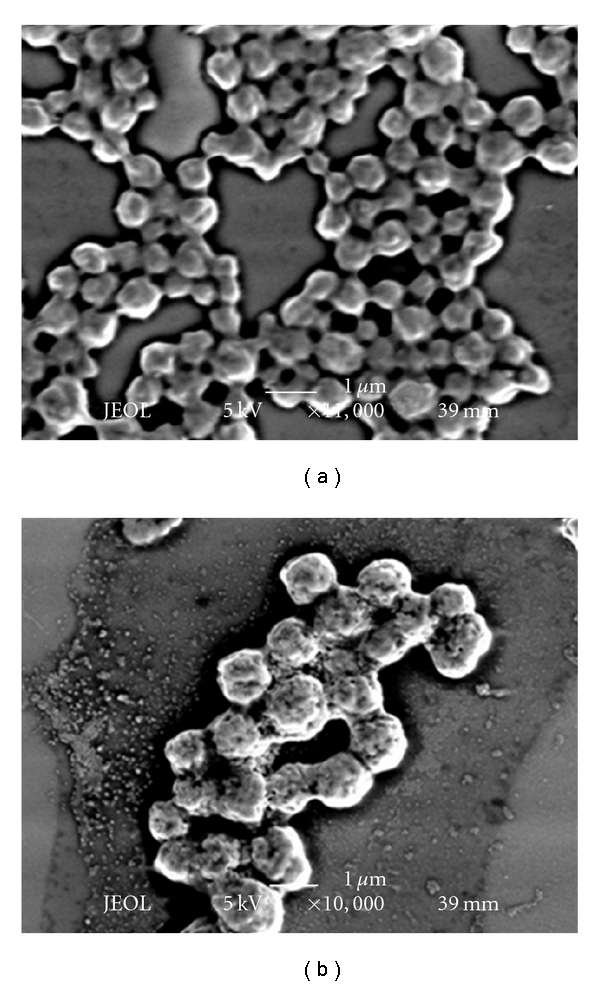
SEM images of (a) PVA/DNA complex (PVA: 1.0 w/v%) and (b) PVA/HAp/DNA complex (PVA: 1.0 w/v%, HAp: 0.1 w/v%) obtained by high hydrostatic pressurization (980 MPa, 10 min, 40°C). DNA conc.: 0.0025 w/v%.

**Figure 2:**
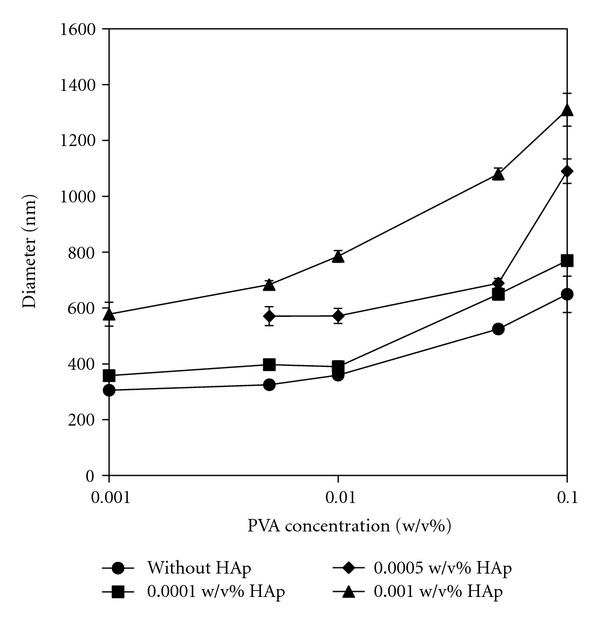
DLS measurement of PVA/DNA and PVA/HAp/DNA complexes at various PVA and HAp concentrations. DNA conc.: 0.0025 w/v%. Each value represents the mean ± SD (*n* = 5).

**Figure 3:**
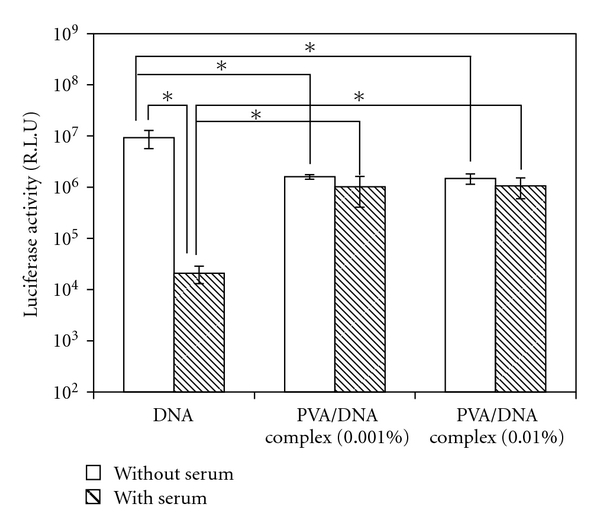
Stability of DNA in PVA/DNA complexes in the presence of serum. Each value represents the mean ± SD (*n* = 3). *P < .05.

The mixture solution of PVA, Hap, and DNA was hydrostatically pressurized at 980 MPa and 40°C for 10 min using a high hydrostatic pressure machine. After removal of pressure, the obtained PVA/HAp/DNA complexes were observed by SEM.  Figure 1 shows typical SEM images of PVA/DNA (PVA: 1.0%) and PVA/HAp/DNA complexes (PVA: 1.0%, HAp: 0.1%). Many particles less than 1 *μ*m were observed for the PVA/DNA complex. The surface of PVA/DNA particles was smooth. On the other hand, in the case of PVA/HAp/DNA complexes, irregular particle surfaces were observed without any significant HAp absorption on the particles, showing that HAp particles were encapsulated in the PVA/HAp/DNA complexes. When excess HAps were mixed with PVA and DNA, many aggregates of HAps on the PVA/HAp/DNA particles obtained by the pressurization were clearly visible (data not shown). The particle size of PVA/DNA and PVA/HAp/DNA complexes at various concentrations of PVA and HAp were measured by DLS measurement (Figure 2, Table 1). The diameter of PVA/DNA particles without HAp increased with increased PVA concentration, which corresponds to our previous report [1–4]. This tendency was exhibited for the particle size of PVA/HAp/DNA complexes, irrespective of HAp concentration. At each PVA concentration, the diameter of PVA/HAp/DNA particles increased with increased HAp concentration, indicating that HAp particles were significantly encapsulated in PVA/HAp/DNA complexes at these concentrations of PVA and HAp. From these results of SEM observation and DLS measurement, it was clear that nano-, microscaled composites of PVA, Hap, and DNA were obtained by high hydrostatic pressurization, and the size of PVA/HAp/DNA particles depended on PVA and HAp concentrations. To investigate the stability of DNA in the PVA/DNA particles on serum condition, PVA/DNA particles were incubated in medium containing 10% serum for 20 h, and then subjected to in vitro transcription and translation (Figure 3). The high luciferase activity of DNA was showed on the condition without serum, whereas the luciferase activity was remarkably reduced after incubation with serum. On the other hand, there is no difference in the luciferase activity of DNA in PVA/DNA particles before and after incubation with serum, indicating the high stability of DNA in PVA/DNA particles against serum. 

To date, many methods for preparation of composite materials of PVA and HAp, such as in situ crystallization of HAp in PVA hydrogel [28], gelation of PVA solution mixed with HAp crystals [29], and alternating soaking reaction, which promote HAp crystallization on/in gel [30], have been reported. Large-scaled composite hydrogels (several centimeters) have been prepared for use in biomedical applications such as cartilage and bone. However, few preparation methods of nanocomposites of PVA and Hap have been reported. In this study, the nano-, microparticles of PVA, HAp and DNA were obtained by using high hydrostatic pressure technology. It is thought that this is achieved by the pressure-induced quick formation of PVA particles that could incorporate secondary and third substrates, such as DNA and HAp, without phase separation [15, 31].

### 3.2. Cytotoxicity Test

**Figure 4:**
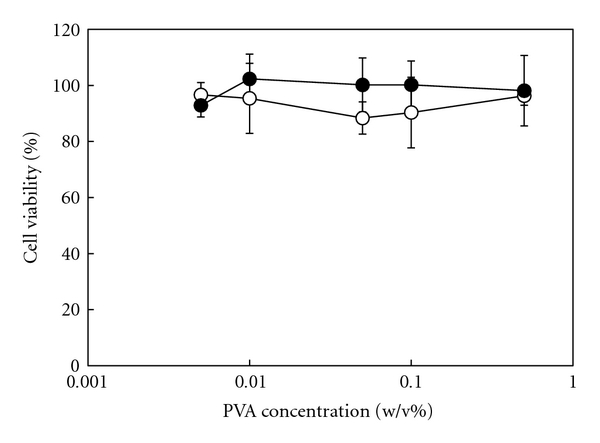
Viability of COS-7 cells incubated with (white) PVA/DNA complexes and (black) PVA/HAp/DNA complexes for 24  h. DNA conc.: 0.0025 w/v%. Each value represents the mean ± SD (*n* = 3).

Figure 4 shows the result of the cytotoxicity test of PVA/DNA and PVA/HAp/DNA complexes. The high viability of COS-7 cells incubated with them is shown, irrespective of the concentration of PVA and HAp. PVA and HAp are biocompatible materials [32, 33]. The PVA/DNA complex is nontoxic because of the composite formation of PVA and DNA via hydrogen bonding interaction [16]. HAps were encapsulated in PVA/HAp/DNA complexes. Consequently, it is considered that the nontoxicity of PVA/HAp/DNA complexes was achieved by these combinations.

### 3.3. Cellular Uptake of PVA/HAp/DNA Nanoparticles

**Figure 5:**
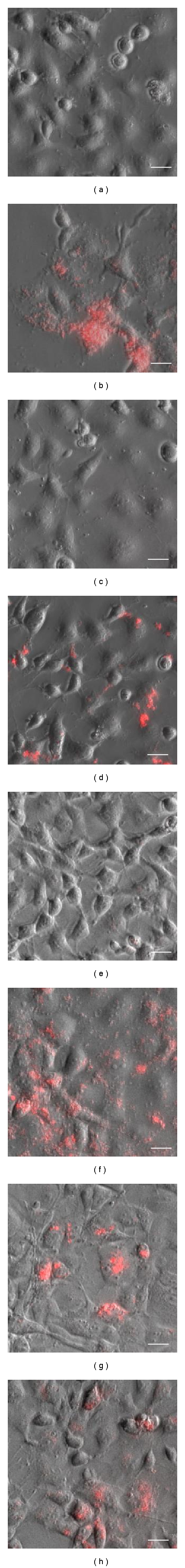
Fluorescent microscopic images of COS-7 cells incubated with (a, e) DNA, (b, f) HAp/DNA complex, (c, g) PVA/DNA complex, and (d, h) PVA/HAp/DNA complex for (a–d) 1 h and (e–h) 24 h. Scale bars are 10 *μ*m.

In order to investigate cellular uptake of the HAp/DNA complex, PVA/DNA, and PVA/HAp/DNA nanoparticles, rhodamine-labeled plasmid DNA was used. Figure 4 shows fluorescent microscopic images of COS-7 cells incubated with complexes of PVA, Hap, and rhodamine-labeled DNA for one and 24 h. After 1 h incubation, fluorescent spots were poorly observed for DNA and PVA/DNA nanoparticles (Figures 5(a) and 5(c)), whereas a lot of bright red fluorescent spots on many cells were shown in the case of HAp/DNA and PVA/HAp/DNA complexes (Figures 5(b) and 5(d)), indicating the effective absorption of them onto cells because of their higher specific gravity. However, strong aggregation of HAp/DNA complexes was observed due to the fact that the nature of HAp particles tends to result in an aggregation in the aqueous medium [34]. For PVA/HAp/DNA nanoparticles, PVA bearing HAp could attenuate the aggregation property of HAp. After 24 h incubation, the aggregation of the HAp/DNA composite was still observed (Figure 5(f)). The internalization of PVA/HAp/DNA nanoparticles into cells was exhibited. Also, the subcellular distribution of DNA was observed in some cells (Figure 5(h)) similar to that of PVA/DNA nanoparticles (Figure 5(g)). This strongly suggests that HAp in PVA/HAp/DNA nanoparticles could be dissolved during the intracellular process, probably due to the endocytosis pathway.

### 3.4. In Vitro Transfection Using PVA/HAp/DNA Nanoparticles

**Figure 6:**
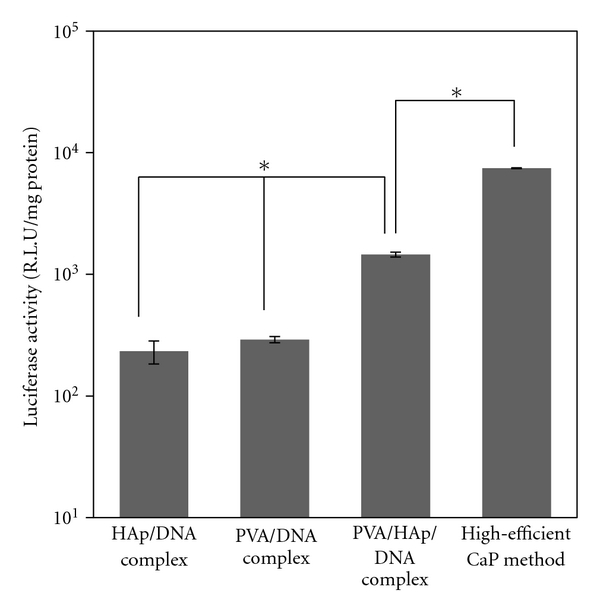
*In vitro* transfection using HAp/DNA, PVA/DNA and PVA/HAp/DNA complexes. Each value represents the mean ± SD (*n* = 3).   *P < .05.

The expressing of the delivered DNA compositing with PVA and HAp was assayed by measuring luciferase activity (Figure 6). Low luciferase activity was shown for the HAp/DNA complex. This is caused by the strong aggregation of HAp/DNA complexes [20]. The level of luciferase activity of PVA/DNA nanoparticles was similar to that of the HAp/DNA complex due to the slow internalization of PVA/DNA nanoparticles into cells, which could probably permit DNA degradation. In the case of the PVA/HAp/DNA nanoparticles, which can be taken up by cells quickly, high luciferase activity was shown, indicating that the encapsulation of HAp in PVA/DNA nanoparticles could enhance the transfection efficiency in vitro. However, the transection efficiency of the PVA/HAp/DNA nanoparticles was lower than in the high-efficient calcium phosphate transfection method, which is optimized for in vitro transfection [21]. 

### 3.5. In Vivo Transfection Using Hydrodynamic Injection

**Figure 7:**
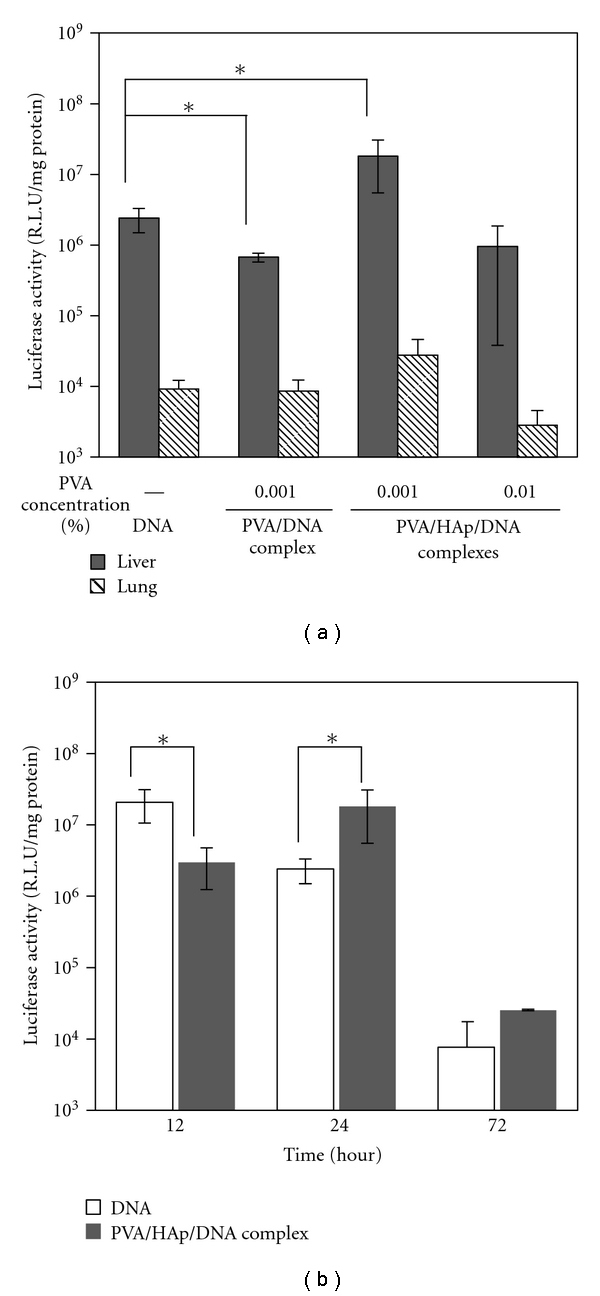
Transgene expression (luciferase activity) of plasmid DNA, PVA/DNA, and PVA/HAp/DNA complexes injected by *in vivo* hydrodynamic method. (b) Time course of transgene expression of plasmid DNA and PVA/HAp/DNA complexes injected by *in vivo* hydrodynamic method. Each value represents the mean ± SD (*n* = 3).  *P < .05.

*In vivo* transfection was performed by using a hydrodynamic method (Figure 7). This method is known as an effective plasmid DNA transfection method without gene carrier to liver [35]. Figure 7(a) shows the results of *in vivo* hydrodynamic injection using various nanoparticles. The luciferase activity of the PVA/DNA complex (PVA: 0.001 w/v%) was lower than that of DNA injection, whereas high luciferase activity was achieved for PVA/HAp/DNA nanoparticles at the PVA concentration of 0.001 w/v% (HAp: 0.0001 w/v%). At PVA concentration of 0.01 w/v% (HAp: 0.001 w/v%), the luciferase activity of PVA/HAp/DNA nanoparticles decreased compared to that of 0.001 w/v%. This is thought to be caused by the insignificant uptake of the large particles of PVA/HAp/DNA nanoparticles (about 780 nm, Figure 2, Table 1) by hepatocytes [36]. When the luciferase activity in lung was also investigated, the low activity was detected in lung compared to that in liver, irrespective of type of nanoparticles. 

The time-course of transgene activity was also investigated (Figure 7(b)). For plasmid DNA, the highest value for luciferase activity was detected after 12 hours, and the level of gene expression significantly decreased over time. On the other hand, in the case of PVA/HAp/DNA nanoparticles, the highest value for luciferase activity was achieved for 24 hours. This result indicates that the PVA/HAp/DNA nanoparticles could prolong the gene expression. We assumed that PVA/HAp/DNA nanoparticles could be accumulated due to the relative high stability, which are continuously transcribed and translated (Figure 3).

## 4. Conclusion

We successfully developed PVA/DNA nanoparticles encapsulating HAps by using simple high hydrostatic pressure technology. They could enhance the transfection efficiency without any significant cytotoxicity *in vitro* and *in vivo* hydrodynamic injection. Consequently, the potential use of HAp could be expected as an enhancer of gene transfer activity of PVA/DNA nanoparticles.

## Figures and Tables

**Table 1:** DLS measurement of PVA/DNA and PVA/HAp/DNA complexes at various PVA and HAp concentrations. DNA conc.: 0.0025 w/v%.

HAp (%)	Average diameter (nm) (PDI) PVA (%)				
	0.001	0.005	0.01	0.05	0.1
0	306 ± 7 (0.08 ± 0.02)	325 ± 8 (0.13 ± 0.02)	360 ± 11 (0.16 ± 0.02)	525 ± 16 (0.07 ± 0.03)	649 ± 65 (0.11 ± 0.02)
0.00001	346 ± 4 (0.12 ± 0.02)	393 ± 8 (0.13 ± 0.02)	355 ± 14 (0.20 ± 0.02)	602 ± 17 (0.08 ± 0.03)	756 ± 17 (0.12 ± 0.03)
0.0001	358 ± 3 (0.11 ± 0.03)	397 ± 8 (0.10 ± 0.03)	390 ± 8 (0.19 ± 0.01)	649 ± 21 (0.12 ± 0.03)	770 ± 20 (0.08 ± 0.03)
0.0005		571 ± 34 (0.24 ± 0.13)	572 ± 27 (0.15 ± 0.03)	689 ± 17 (0.05 ± 0.03)	1090 ± 44 (0.21 ± 0.03)
0.001	578 ± 42 (0.278 ± 0.04)	684 ± 14 (0.11 ± 0.21)	785 ± 21 (0.11 ± 0.02)	1080 ± 21 (0.25 ± 0.02)	1310 ± 59 (0.23 ± 0.03)
